# Environment shapes gut microbiome and determines susceptibility to DSS-colitis in *Adamdec1^-/-^* mice

**DOI:** 10.3389/fimmu.2026.1824007

**Published:** 2026-07-27

**Authors:** Tomoko Kumagai, Silke Rath, Shuangqi Fan, Farooq Z. Rahman, Andrew M. Smith

**Affiliations:** 1 Department of Gastroenterology, Whipps Cross University Hospital, Barts Health NHS Trust, London, United Kingdom; 2 Department of Microbial Diseases, University College London (UCL) Eastman Dental Institute, University College London, London, United Kingdom; 3 College of Veterinary Medicine, South China Agricultural University, Guangzhou, China; 4 Gastrointestinal Services, University College London Hospitals NHS Foundation Trust, London, United Kingdom

**Keywords:** ADAMDEC1, gene-environment interactions, gut microbiome, mucosal inflammation, phenotype variability

## Abstract

**Background:**

Inflammatory bowel disease (IBD) is characterized by marked clinical heterogeneity, reflecting complex interactions between host genetics, environmental exposures, and the gut microbiome. Although over 300 genetic risk loci have been identified, these account for only a fraction of disease variance, highlighting the importance of gene–environment interactions. ADAM-like Decysin-1 (ADAMDEC1) is a gastrointestinal-restricted metalloprotease implicated in mucosal repair and immune regulation, with reduced expression reported in IBD. While *Adamdec1*-deficient mice exhibit increased susceptibility to colitis, the mechanisms underlying phenotypic variability remain poorly understood.

**Methods:**

To dissect genetic and environmental contributions to colitis severity, C57BL/6J wild-type (WT) and *Adamdec1^−/−^* mice were co-housed in two distinct environments and subjected to dextran sodium sulfate (DSS)-induced colitis. Disease severity was assessed by weight loss, immune cell infiltration, and transcriptional profiling of inflammatory and epithelial repair markers using flow cytometry and qPCR. Gut microbiome composition was analyzed from fecal samples collected following extended co-housing to evaluate environment-driven microbial differences.

**Results:**

Environmental factors exerted a stronger influence than genotype on gut microbiome composition. WT mice displayed consistent inflammatory and transcriptional responses to DSS across environments, whereas *Adamdec1*^−/−^ mice exhibited marked cage-dependent variability, ranging from severe colitis to a mild, WT-like phenotype. Severe disease in *Adamdec1*^−/−^ mice was associated with enhanced neutrophil recruitment, increased CD11b expression, altered monocyte/macrophage activation, impaired epithelial proliferation, and disrupted stem cell–associated gene expression. These phenotypic differences correlated with distinct microbiome profiles indicating genotype-dependent microbial pathogenicity.

**Conclusion:**

These findings demonstrate that loss of *Adamdec1* creates a state of heightened sensitivity to environmental and microbiome related factors, in which gut microbial composition correlates with differences in inflammatory and epithelial outcomes during colitis. This study identifies a gene–environment–microbiome axis associated with phenotypic variability in intestinal inflammation and provides mechanistic insight into the heterogeneity of IBD. Targeting environmentally driven microbial modifiers may represent a promising strategy for personalized intervention in genetically susceptible individuals.

## 1 Introduction

The dysregulation of the mucosal immune system leading to chronic intestinal inflammation, as seen in inflammatory bowel disease (IBD), is thought to result from a complex interplay between environmental factors, the gut microbiome, and the immune system in genetically susceptible individuals. Although numerous disease-associated genetic variants have been identified in IBD, they exhibit incomplete penetrance and variable genotype expressivity (1). Environmental factors are believed to modulate this variability, as evidenced by findings from monozygotic twin and migration studies (2–6).

Dysbiosis of the gut microbiome plays a well-established role in IBD and numerous other human diseases (7–10). While it remains unclear whether dysbiosis is a cause or consequence of disease, studies in mouse models have demonstrated that the microbiome can directly influence the host immune system, suggesting a causative role in the pathogenesis of intestinal mucosal inflammation (11–13). Environmental factors, including diet, medications, hygiene, and social stress, have been shown to alter gut microbiome composition (14–17). Indeed, environmental influences are estimated to account for approximately 22–36% of the variability in the human gut microbiome, whereas host genetics contribute only 1.9–8.1% of interpersonal microbiota variation (18). Thus, environmental factors appear to exert a substantial influence on microbiome composition. In mouse models, cage effects have been shown to have a greater impact on bacterial microbiome composition than either genetic background or sex, accounting for up to 31.7% of the observed variance, even when animals are maintained under otherwise identical housing conditions (19). Such effects can generate considerable inter-cage microbiome variability. Factors including coprophagy, stochastic microbial drift, transmission of opportunistic microbes, and subtle differences in cage environment such as bedding-types or bedding-change frequency are thought to contribute to these cage-dependent differences in microbiome composition (19–22). ADAM-like DECysin 1 (ADAMDEC1) is a highly conserved metalloprotease expressed predominantly in the gastrointestinal (GI) tract under healthy conditions across multiple mammalian species, including humans. Although its physiological function and catalytic substrates remain undefined, reduced expression of ADAMDEC1 has been observed in the intestinal tissues of individuals with IBD and colorectal cancer, suggesting a vital role in GI homeostasis and potentially in disease pathogenesis (23–25). ADAMDEC1 was originally identified as a macrophage and dendritic cell specific metalloprotease, which was upregulated in response to bacterial ligands and during monocyte-to-macrophage maturation in lamina propria (25–28). However, more recent studies using single cell transcriptomic analysis of intestinal cells have identified its expression in a subset of colonic fibroblasts within the bowel (29, 30). These ADAMDEC1-expressing fibroblasts produce high levels of immune-related chemokines, indicating a potential role in modulating inflammation within the colonic lamina propria (30, 31).

Previous studies using *Adamdec1* knockout (*Adamdec1*^−/−^) mice have shown heightened mucosal inflammation following dextran sodium sulfate (DSS)-induced colitis, further supporting a protective role for ADAMDEC1 in intestinal inflammation (25, 30). In these models, loss of *Adamdec1* was associated with increased epithelial injury and abnormal extracellular matrix remodeling. Additionally, *Adamdec1* expression was upregulated in a subpopulation of fibroblasts in wild-type (WT) mice during DSS-induced colitis (30). These cells, located near the crypt base, exhibited gene expression profiles indicative of roles in epithelial proliferation and differentiation.

To date, the relationship between ADAMDEC1 and the gut microbiome has not been explored. Given the proposed role of ADAMDEC1 in intestinal immune regulation and maintenance of mucosal homeostasis, it is plausible that loss of ADAMDEC1 may influence host–microbiome interactions within the gut. Determining whether ADAMDEC1 deficiency alters microbiome composition or modifies host responses to microbial communities may therefore provide important insight into mechanisms underlying intestinal inflammation and disease susceptibility. Accordingly, this study aimed to investigate how variation in *Adamdec1* expression influences the severity of DSS-induced colonic inflammation, and whether the heightened inflammatory response observed in *Adamdec1*-deficient mice is linked to alterations in the gut microbiome.

## 2 Materials and methods

### 2.1 Animals

Animal studies were performed in accordance with the UK Animals (Scientific Procedures) Act 1986 and European Directive 2010/63/EU on the protection of animals used for scientific purposes.

#### 2.1.1 Sources and generation of *Adamdec1*^-/-^ and WT mice

*Adamdec1* heterozygous mice were generated by targeted mutagenesis of the *Adamdec1* gene 1227 on chromosome 8 and insertion of a neomycin resistant cassette. The line of *Adamdec1* heterozygous mice was reconstituted from frozen embryonic stem cells re-derived from 129/OlaHsd mice embryos at the Deltagen repository (Deltagen, Inc, USA). The chimeric mice were backcrossed onto C57BL/6 mice for at least 6 generations before three *Adamdec1* heterozygous mice were purchased. Heterozygous mice were crossed to generate WT and *Adamdec1*^-/-^ mice. Genotyping was performed by polymerase chain reaction (PCR) using *Adamdec1* wild-type gene-specific primers: AGCTTGAGCGCAAACCCAATGCTTC and CCTCAGGTACTGATTCATCACACAG (322bp), and *Adamdec1^-/-^* mice primers: GACGAGTTCTTCTGAGGGGATCGATC and CCTCAGGTACTGATTCATCACACAG (600bp) (**Supplementary Figure 1**).

#### 2.1.2 Husbandry of mice

Mice were bred and maintained in specific-pathogen-free, individually ventilated cages in the animal facility of the University College London (UCL) Biological Sciences Unit (BSU). During the stock maintenance, the litters were weaned from their parents at the age of 4–5 weeks and moved into new cages separated by sex with a maximum of 5 mice per cage. The environmental conditions such as temperature, humidity and hours of light (standard 12 hours light/12 hours dark cycle) were kept constant. The cages and bedding were changed weekly. Mice were fed, following standard BSU guidelines, with sterilized Harlan 2018 Teklad Global 18% protein rodent diet, and breeders were fed with sterilized 19% protein rodent diet. Drinking water was sterilized. Routine health screens for infection were carried out for parasites and opportunistic infections.

### 2.2 Cohousing experiment

Twenty female WT and *Adamdec1^-/-^* mice were housed per litter and genotype from birth until the age of 10 weeks in six individual cages. Subsequently, mice were assigned to a cage irrespective of genotype into two larger cages (Group size 8 and 12). Mice were cohoused for a further 8 weeks before being exposed to DSS. A female-only cohort was used for the co-housing experiment to comply with UK Animals (Scientific Procedures) Act 1986 guidelines, as co-housing larger numbers of adult male mice is not permitted due to the increased risk of aggression.

### 2.3 Dextran sodium sulfate-induced colitis

In the initial experiment, mixed-sex mice at 7–8 weeks old were housed per genotype per sex and given 2% DSS (molecular weight 36,000–50,000; MP Biomedicals, Cambridge, UK) in drinking water for seven days, as previously described (22), and weighed daily. In the co-housing experiment, 18–19-week-old female mice received the same DSS treatment. Mice were weighed daily and sacrificed on day 9 for tissue collection.

### 2.4 Fecal sample collection

Cages were individually placed in a biosafety cabinet; the mice were individually transferred to an empty autoclaved cage and allowed to defecate normally. Three to four fecal pellets from each mouse were collected using sterile forceps, placed in a sterile 1.5 ml tube and stored at -80 °C until DNA extraction took place. All samples were collected in the morning between 7am and 10am.

### 2.5 Gut bacterial microbiome analysis

#### 2.5.1 DNA sequencing of murine fecal samples

Microbial DNA from the murine fecal samples was extracted using QIAamp PowerFecal Pro DNA Kit (Qiagen) following the manufacturer’s protocol. Library preparation and sequencing of the amplicons were performed by UCL Genomic department. By using the Swift Amplicon 16S+ITS panel (Swift Biosciences), all variable 16S rRNA gene regions were amplified by multiplex PCR according to the manufacturer (An initial 98 °C step for 30 seconds was followed by 4 cycles of denaturation at 98 °C for 10 seconds; annealing at 63 °C for 5 minutes; and an extension step at 65 °C for 1 minute. A second amplification for 22 cycles (denaturing at 98 °C for 10 seconds followed by 64 °C for 1 minute and 65 °C for 1 minute) was conducted.). Sequencing of the equi-volume pooled PCR amplicons was performed using the Illumina Miseq with a v2–500 cycle run.

The 16S rRNA gene sequencing data was analyzed using the open-source pipeline 16S SNAPP (https://github.com/swiftbiosciences/16S-SNAPP-py3) which involves the following steps. First, short and low-quality reads as well as primers were removed using Cutadapt (Version 3.5), and amplicon sequence variants (ASVs) were identified using DADA2 pipeline (Version 1.16). Duplicate reads and chimeras within the ASVs were identified and removed using VSEARCH (Version 2.16.0). The ASVs were then searched and mapped to the reference 16S rRNA nucleotide sequence database Ribosomal Database Project (RDP) (Version 11.5), using the alignment searching tool BLAST (Version 2.2.30). The mapped sequences were then computed through Python 3 (Version 3.8.6) to generate a consensus sequence of the amplified 16S rRNA gene regions for each bacterium. These sequences were then classified using the RDP classifier (Trained classifier version 2.13) at the species level in order to generate an abundance lineage table.

#### 2.5.2 Beta diversity

β-diversity was analyzed in the PRIMER-e (Plymouth Routines in Multivariate Ecological Research) software (Version 7.0.13). For this, the Bray-Curtis distance was used to measure the difference in ASV composition between samples. Non-metric multi-dimensional scaling (nMDS) plots were created based on Bray-Curtis similarity of the square root-transformed relative abundance data. Permutational analysis of variance (PERMANOVA) was used to determine if the observations in the β diversity were statistically significant.

#### 2.5.3 Alpha diversity

Chao1 and Shannon indices were applied to the ASVs to determine the α-diversity using the R package vegan (Version 2.5-7). Change in the α-diversity between the pre-pooling post-cohousing samples were calculated using paired t-test by GraphPad Prism (version 9.3.1 for Windows; Graph-Pad Software).

#### 2.5.4 Relative abundances

Statistical differences in the relative abundances of bacteria were determined at multiple taxonomic levels by Kruskal-Wallis tests using GraphPad Prism (version 9.3.1 for Windows; Graph-Pad Software).

### 2.6 Colonic cell population and gene expression analysis

Mice were culled and the colons were harvested. Colon length was measured as a macroscopic indicator of disease severity. The cecum from each sample was removed and discarded. The distal 1.5 cm of each colonic sample were removed and placed in 500 μl of RNAlater (Qiagen) and stored at -80 °C for subsequent quantitative polymerase chain reaction (qPCR) analysis.

#### 2.6.1 Flow cytometry

Colonic lamina propria cells were isolated using the method described previously (32). The cells were stained with Live/Dead stain (Thermo Fisher Scientific) and blocked with Fc block (BioLegend) before staining with CD45 PE-Cy7 (BioLegend 103113), CD3 FITC (BioLegend 100203), CD11b PE (BioLegend 149005), CD11c BV786 (BD Biosciences 565587), MHCII Alexa Fluor 647 (BD Biosciences 562367), Ly6C BV711 (BioLegened 128037), Ly6G BV421 (BioLegend 127628), F4/80 PE/CF594 (BioLegend 123145), CX3CR1 PE (BioLegend 149005), and fixed with 4% paraformaldehyde. Cells run on a BD LSR Fortessa (BD Biosciences) were analyzed using FlowJo 10.7.2 (BD Biosciences). For mean fluorescence intensity (MFI)-based analyses, WT animals were used as internal controls within each cage to account for batch effects. Accordingly, MFI values for *Adamdec1^-/-^* mice were normalized to their respective WT controls within the same cage.

#### 2.6.2 qPCR

Total RNA from mouse colonic samples were extracted using Qiagen RNeasy Mini Kit (Qiagen). Colonic samples collected for the qPCR analysis were thawed and RNAlater was discarded. After washing with PBS, the samples were submerged in RLT buffer, containing 1% of 2-mercaptoethanol. Then 7 mm diameter stainless steel Tungsten Carbide Beads (Qiagen) were added to each sample, and the colonic tissues were homogenized using TissueLyzer LT (Qiagen) at speed of 50 Hz. RNA was extracted using the manufacturer’s protocol. Total RNA was converted to complementary DNA (cDNA) using M-MLV reverse transcriptase (Promega) and C1000 Touch™ Thermal Cycler (BioRad). qPCR was performed using the SYBR^®^ Green Real-Time PCR Kit (Qiagen) on a Mastercycler^®^ep realplex (Eppendorf). The primers for the genes examined are listed in **Table 1**. The expressions of *Actb* and *GAPDH* were tested and were stable across the samples, thus, these two genes were used as housekeeping genes. One-way ANOVA test was performed to determine whether the differences observed were statistically significant.

**Table 1:** Sequences of the qPCR primers used to investigate gene expression of the mouse colonic samples.

Target gene	Forward primer (5’ to 3’)	Reverse primer (5’ to 3’)
*Actb*	GGGCCGCGTCTCCTTT	ATCCTTTCTCTCCAGTGCTCAGA
*Gapdh*	AAGGTCATCCCAGAGCTGAA	CTGCTTCACCACCTTCTTGA
*Cxcl1*	TCCAGAGCTTGAAGGTGTTGCC	AACCAAGGGAGCTTCAGGGTCA
*Il-1β*	TCAACAAGATAGAAGTCAAGAGCAA	ATGGTGAAGTCAATTATGTCCTGA
*Tnf-α*	CCACCACGCTCTTCTGTCTA	AGGGTCTGGGCCATAGAACT
*Ccl2*	GCTACAAGAGGATCACCAGCAG	GTCTGGACCCATTCCTTCTTGG
*Ccr2*	CCTTGGGAATGAGTAACTGTGTGAT	ATGGAGAGATACCTTCGGAACTTCT
*Mki67*	AGAGCCTTAGCAATAGCAACG	GTCTCCCGCGATTCCTCTG
*Fgf2*	GGCTGCTGGCTTCTAAGTGTGT	GCCCAGTTCGTTTCAGTGCC
*Lgr5*	TCTTCACCTCCTACCTGGACCT	GGCGTAGTCTGCTATGTGGTGT

### 2.7 Statistical analysis

Statistical significance was determined by two-tailed unpaired t-test, paired t-test, repeated measures ANOVA, one-way ANOVA, Mann-Whitney or Kruskal-Wallis test using GraphPad Prism (version 9.3.1 for Windows; Graph-Pad Software). Statistical significance for PERMANOVA (i.e., permutation-based p-value) was calculated using the default options in the package PERMANOVA+ for PRIMER. Results are considered significant when *p* < 0.05.

## 3 Results

### 3.1 Increased sensitivity and variability to DSS-induced colitis in *Adamdec1^-/-^* mice

**Figure 1:**
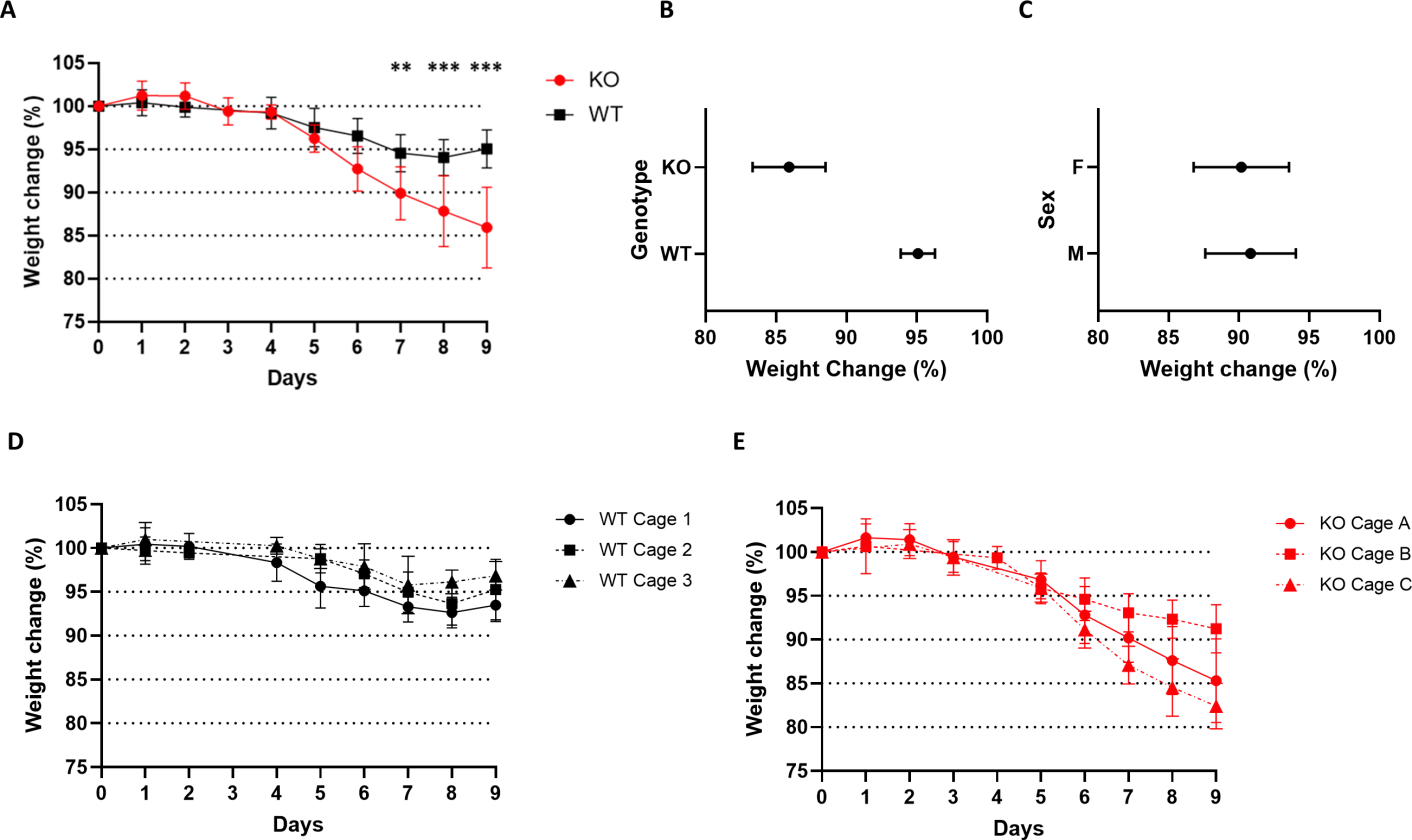
Genotype-, gender-, and cage-dependent effects on DSS-induced weight loss in *Adamdec1^-/-^* and WT mice. **(A)** Weight change in the *Adamdec1^-/-^* and WT mice during 2% DSS challenge. Statistical comparisons were performed using two-way repeated measures ANOVA. **(B)** Weight change in the mice shown as per genotype on day 9 of 2% DSS challenge. **(C)** Weight change in the mice shown as per sex on day 9 of 2% DSS challenge. **(D)** Weight change in WT mice per cage during 2% DSS challenge. **(E)** Weight change in *Adamdec1^-/-^* mice per cage during 2% DSS challenge. All data are shown as mean ± SD. *Adamdec1^-/-^* mouse n=15 (male n = 7, female n = 8), WT mouse n = 15 (male n = 8, female n = 7). KO: *Adamdec1^-/-^*, WT, Wild type; F, Female; M, Male, **: *p* < 0.01, *** *p* < 0.001.

To investigate how loss of *Adamdec1* influences the development of bowel inflammation, *Adamdec1^-/-^* and WT mice, housed per genotype and per sex (aged 7–8 weeks), were challenged with 2% DSS for seven days. Consistent with previous reports (25, 30), *Adamdec1^-/-^* mice exhibited greater weight loss compared to WT controls (**Figures 1A, B**). Notably, *Adamdec1^-/-^* mice also showed markedly higher variability in their response to DSS. The standard deviation of body weight on day 9 was significantly greater in *Adamdec1^-/-^* mice compared to WT mice (4.69 g vs. 2.20 g, *p* = 0.008) (**Figure 1B**), and this variability was not influenced by sex (**Figure 1C**), but appeared to be most strongly influenced by the cage in which the animals were housed (**Figures 1D, E**).

### 3.2 Gut microbiome is shaped by cage environment, not by *Adamdec1* deficiency

To investigate whether the increased variability in DSS susceptibility observed in *Adamdec1^−/−^* mice could be attributed to differences in the gut microbiome, we first analyzed the fecal microbiota of young (aged 4–5 weeks) naïve WT, heterozygous, and *Adamdec1^−/−^* mice. Samples were collected from 16 mice (mixed by sex and genotype) derived from three breeding pairs housed across four cages per littermates (Cages 1-4; group sizes 2-5).

**Figure 2:**
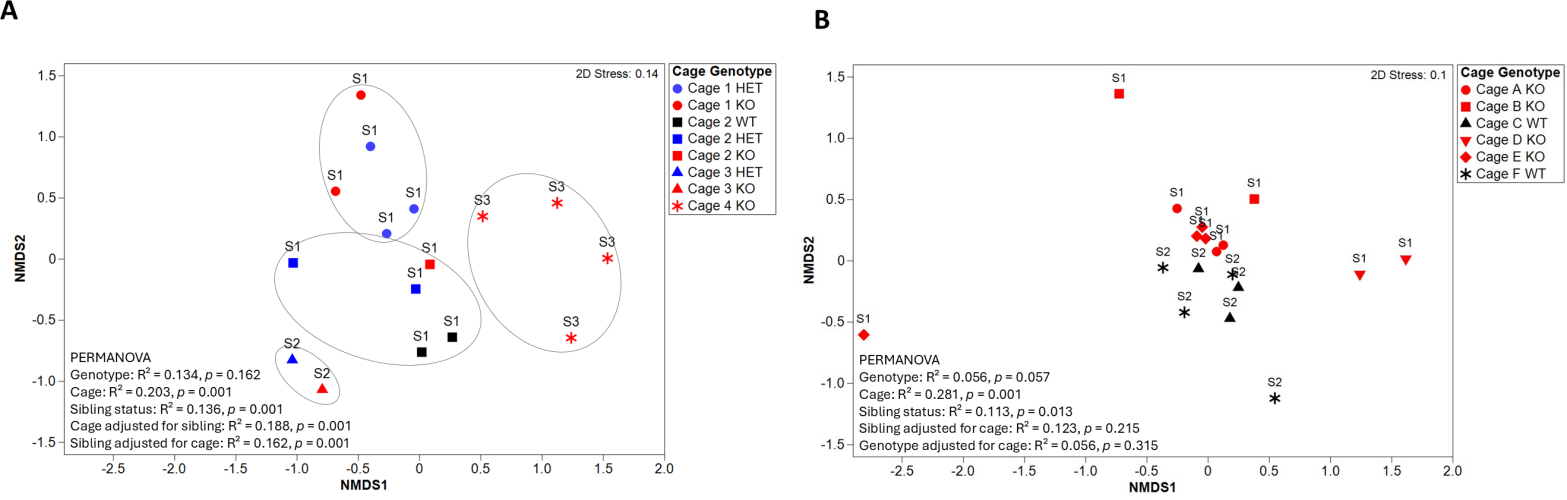
Factors influencing the fecal microbiome composition in mice. Non-metric multidimensional scaling (nMDS) plot showing β-diversity of the murine fecal bacterial microbiome composition. **(A)** Bacterial microbiome composition of *Adamdec1^-/-^* (n = 8), heterozygous (n = 6) and WT (n = 2) mice housed in 4 cages per littermates mixed in genotype and sex. **(B)** Bacterial microbiome composition of *Adamdec1^-/-^* (n = 11) and WT mice (n = 7) when the mice were housed according to their genotype. Genotypes are indicated by color. Individual cages are indicated by shapes. Sibling status (S) is indicated above each mark. KO: *Adamdec1^-/-^*, HET, Heterozygous; WT, Wild type.

Non-metric multidimensional scaling (nMDS) of β-diversity revealed no clustering by genotype, indicating that *Adamdec1* deficiency did not significantly alter fecal microbiome composition (**Figure 2A**). This was supported by PERMANOVA analysis, which showed no genotype effect (*p* = 0.162). In contrast, samples clustered strongly by cage and sibling status, suggesting that microbiome composition was primarily determined by environmental and familial factors. PERMANOVA analysis revealed significant associations of both cage and sibling status with gut microbiota composition (*p* = 0.001 for both). Given the partial overlap between these variables, adjusted PERMANOVA analyses were performed. PERMANOVA analyses accounting for cage and sibling status demonstrated that both variables were independently associated with gut microbiota composition (*p* = 0.001 for both), indicating that each exerted a distinct influence on murine gut microbiota composition.

Previous studies have shown that differences in the gut microbiome between mutant and WT mice are more pronounced when mice are housed separately by genotype, while co-housing leads to convergence of their microbiota (33). To further validate our finding—that microbiome composition is shaped more by cage environment and sibling status than genotype—we analyzed the fecal microbiota of 20 female mice (*Adamdec1^−/−^*, *n* = 12; WT, *n* = 8) housed from birth per litter and genotype in six cages (Cages A-F; group sizes 2-4) for 10 weeks. Only female mice were used, as no significant sex-dependent differences in disease severity were observed in the initial DSS cohort (**Figure 1C**), and to ensure compliance with UK Animals (Scientific Procedures) Act 1986 guidelines for the subsequent co-housing experiment involving larger groups.

As in the previous experiment, nMDS plots demonstrated clustering by cage and sibling status (**Figure 2B**), which were both statistically significant (PERMANOVA: cage *p* = 0.001; sibling state *p* = 0.013). However, after accounting for cage effects in the PERMANOVA analysis, sibling status was no longer a significant determinant of gut microbiota composition (*p* = 0.215), indicating that cage effects accounted for much of the variation previously attributed to sibling status. While some visual clustering by genotype was apparent (**Figure 2B**), this was not statistically significant (PERMANOVA *p* = 0.057). After accounting for cage effects, the association was further attenuated (PERMANOVA *p* = 0.315).

Taken together, these findings demonstrate that in the naïve state, fecal microbiome composition in both *Adamdec1^−/−^* and WT mice is predominantly driven by cage environment, with little evidence of direct genotype-specific effects.

### 3.3 Cage-specific microbiota signatures are plastic and reshaped by cohousing

To further validate that cage environment was the primary determinant of fecal bacterial composition, mice from cages A-F were pooled into two larger cages (Cage I *Adamdec1^-/-^*, n = 5, WT, n = 3 and Cage II *Adamdec1^-/-^*, n = 7, WT, n = 5), mixing genotypes and mice from multiple litters, and co-housed for eight weeks. This allocation was performed at the cage level and was not randomized at the level of individual animals, in order to preserve pre-existing cage-associated microbiota and to monitor changes following co-housing.

**Figure 3:**
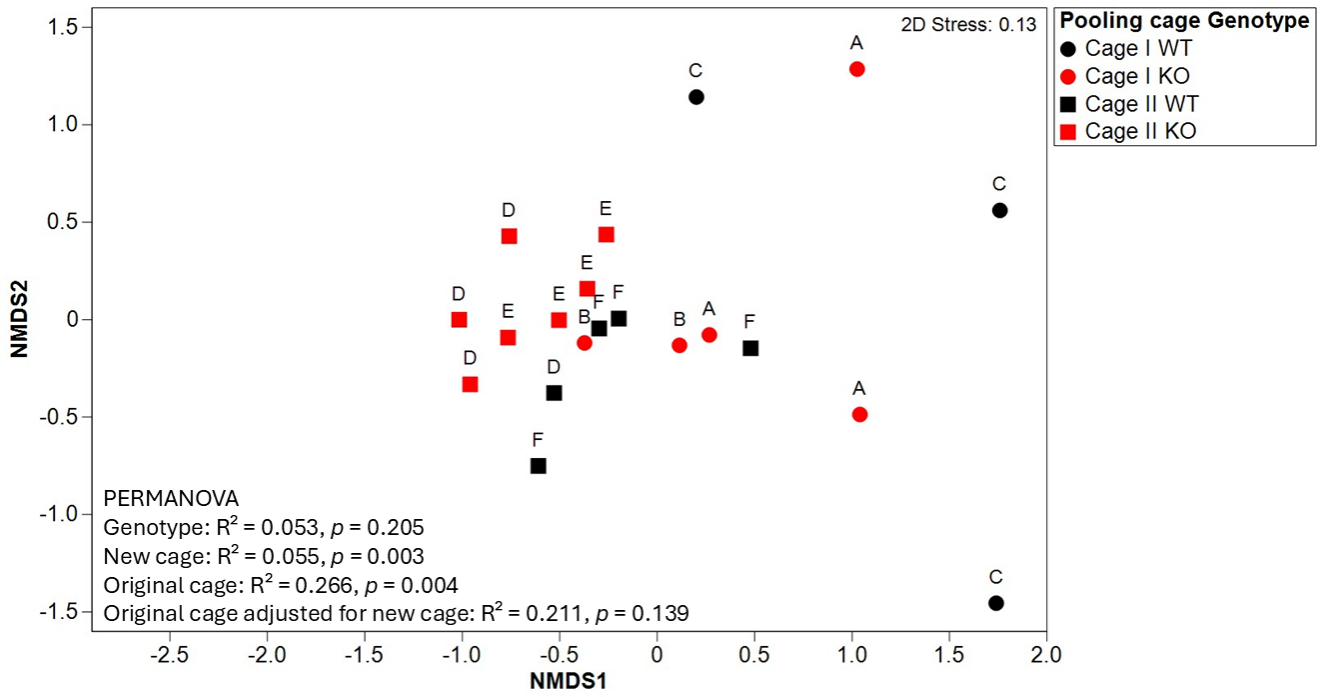
Cage- driven clustering of fecal microbiome composition following long-term cohousing. Non-metric multidimensional scaling (nMDS) plot showing β-diversity of the fecal bacterial microbiome composition in *Adamdec1^-/-^* (n = 12) and WT (n = 8) mice after 8 weeks of cohousing in two larger cages (Cage I and II). Genotypes are indicated by color. Individual cages are indicated by shapes. Original cage (Cage A-F) is indicated above each mark. KO: *Adamdec1^-/-^*, WT. Wild type.

β-diversity analysis of post-cohousing samples revealed clear clustering according to the new pooling cages (**Figure 3**), which was statistically significant (PERMANOVA *p* = 0.003). While the effect of the original cage remained significant in the PERMANOVA analysis (*p* = 0.004), it was no longer significant in the PERMANOVA accounting for the new cage environment (*p* = 0.139), suggesting that the microbiome signature of the original cage had largely been superseded by the influence of the new cage environment.

Genotype continued to have no detectable effect on microbiome composition (PERMANOVA *p* = 0.205), and sibling state no longer influenced β-diversity after pooling (PERMANOVA *p* = 0.426). Importantly, this transition was independent of mouse age (PERMANOVA *p* = 0.295).

**Figure 4:**
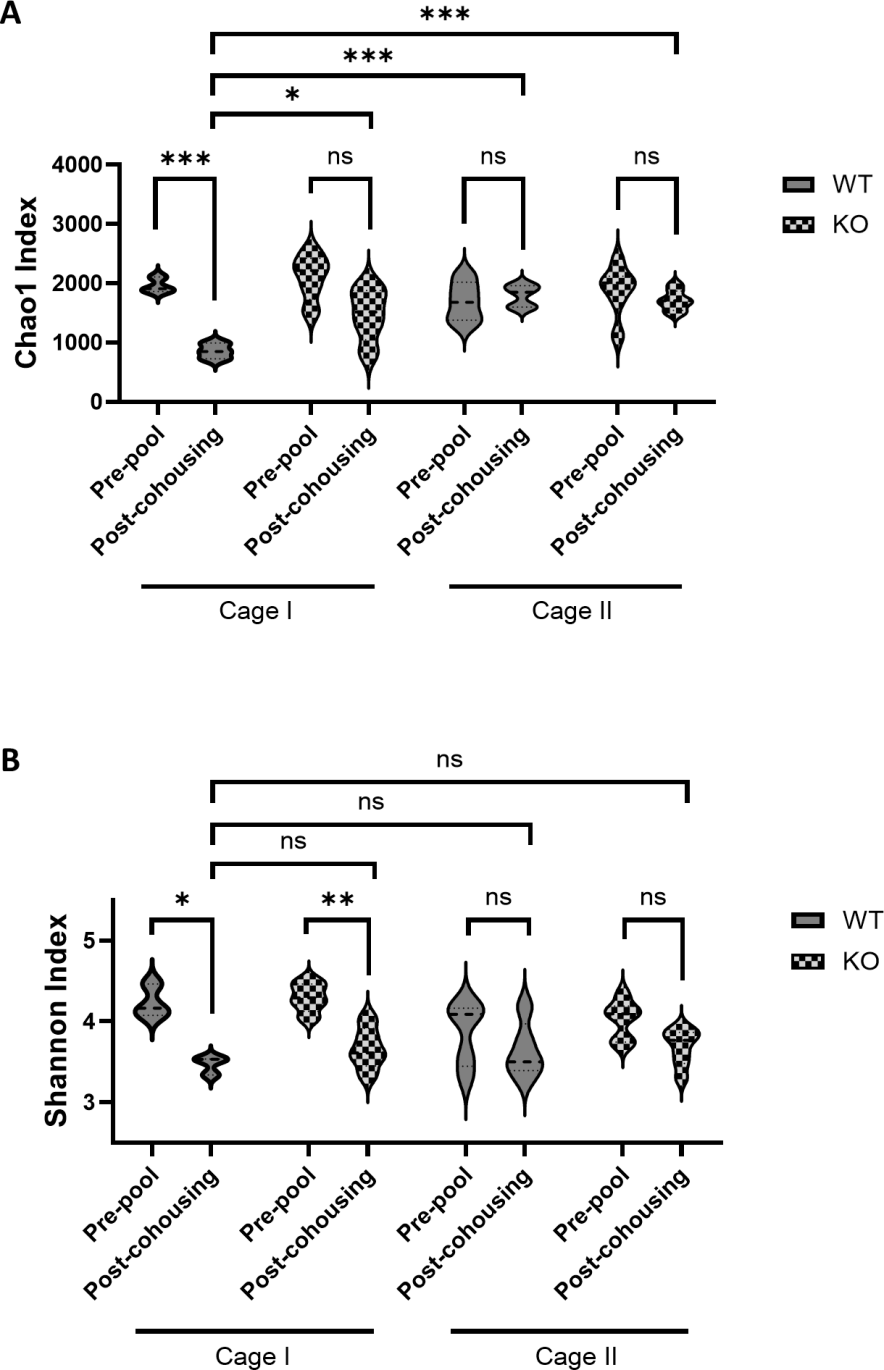
Cage-associated differences in fecal α-diversity following cohousing. **(A)** Chao1 and **(B)** Shannon indices are shown before and after the mice were pooled into Cages I and II. Statistical comparisons between pre-pooling and post-cohousing samples within each cohort were performed using paired *t*-tests. Statistical comparisons between post-cohousing groups were performed using one-way ANOVA. Cage I WT n = 3, Cage II WT n = 5, Cage I *Adamdec1^-/-^* n = 5, Cage II *Adamdec1^-/-^* n = 7. KO: *Adamdec1^-/-^*, WT: Wild type, *p < 0.05, **p < 0.01, ***p < 0.001, ns, Non-significant.

In addition, a reduction in α-diversity of the gut microbiome was observed in the mice housed in Cage I, but not in Cage II (**Figures 4A, B**). Specifically, the Shannon diversity index was significantly decreased in both WT and *Adamdec1^-/-^* mice housed in Cage 1 compared with their respective pre-pooling samples (*p* = 0.022 and *p* = 0.002, respectively) (**Figure 4B**). Species richness, as measured by the Chao1 index, was significantly reduced only in WT mice housed in Cage I by-cohousing (*p* = 0.0004), and was significantly lower compared to the post-cohousing samples of *Adamdec1^-/-^* mice in Cage I and both WT and *Adamdec1^-/-^* mice in Cage II (**Figure 4A**).

Together with the pre-pooling data, these results demonstrate that murine intestinal microbiota composition is predominantly driven by cage environment and that this cage-specific signature is highly plastic. Eight weeks of co-housing was sufficient to erase the microbial patterns established in the original cages and generate a new, distinct composition defined by the new cage environment.

### 3.4 Cage environment drives variability in DSS-induced colitis in *Adamdec1^−/−^* mice

**Figure 5:**
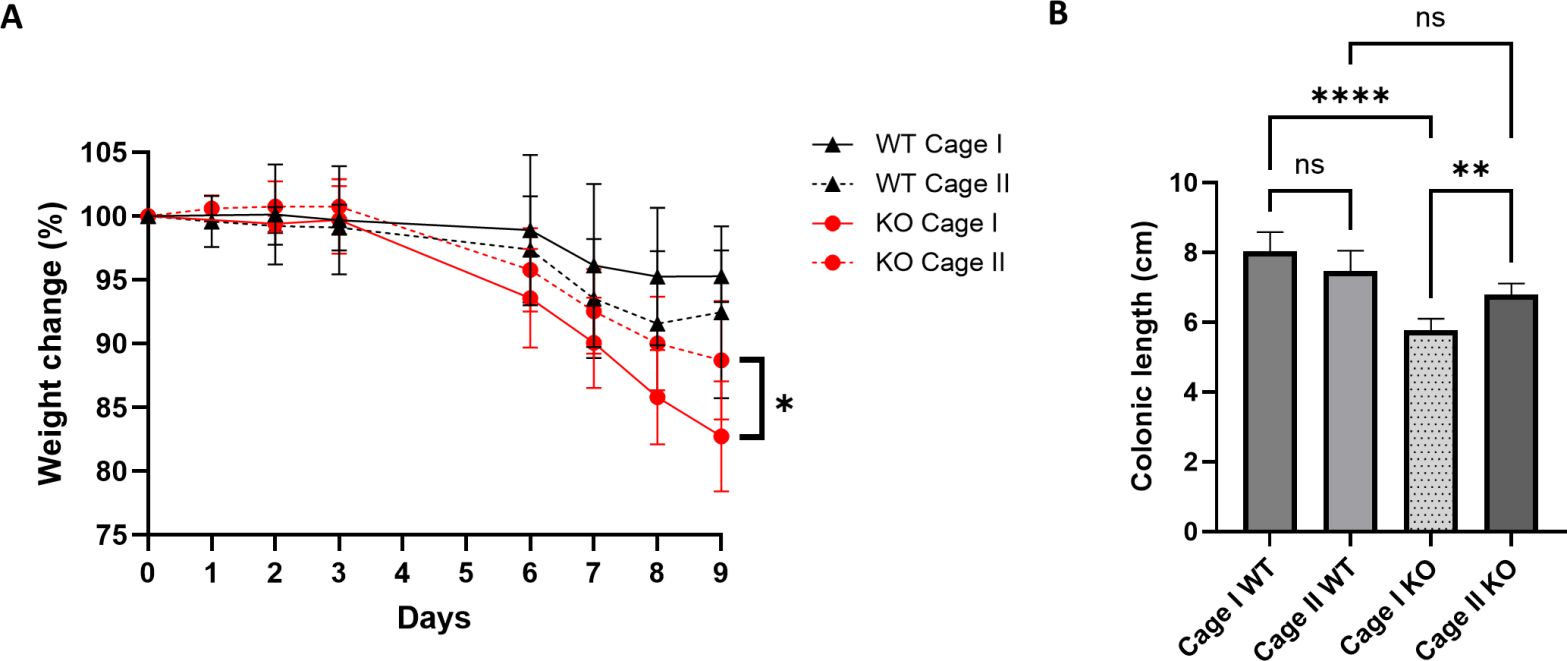
Cage-dependent effects on DSS-induced disease severity in *Adamdec1^-/-^* and WT mice. **(A)** Weight change in the *Adamdec1^-/-^* and WT mice in Cage I and Cage II during 2% DSS challenge. Statistical comparisons were performed using repeated measures ANOVA. **(B)** Length of colon taken from the *Adamdec1^-/-^* and WT mice in Cage I and Cage II on day 9 of 2% DSS challenge. Statistical comparisons were performed using one-way ANOVA. Data are shown as mean ± SD. Cage I WT n = 3, Cage II WT n = 5, Cage I *Adamdec1^-/-^* n = 5, Cage II *Adamdec1^-/-^* n = 7. KO: *Adamdec1^-/-^*, WT, Wild type, *p < 0.05, **p < 0.01, ****p < 0.0001, ns, Non-significant.

The previous DSS experiment involving *Adamdec1^−/−^* and WT mice was conducted with mice housed by genotype (**Figure 1**). To validate our earlier observation of cage-dependent variability in DSS-induced inflammation in *Adamdec1^−/−^* mice, we next challenged the co-housed mice from Cage I and Cage II with DSS, including WT mice as internal controls within each cage. Consistent with our prior findings, *Adamdec1^−/−^* mice again demonstrated cage-dependent variability in response to DSS. Specifically, *Adamdec1^−/−^* mice in Cage I experienced significantly greater weight loss compared to those in Cage II (*p* = 0.047) (**Figure 5A**). This effect appeared specific to the knockout mice: WT mice in Cage I showed the least weight reduction, and no significant difference in weight change was observed between WT mice from the two cages (**Figure 5A**). In addition to the weight findings, a significantly shorter colonic length was observed in the *Adamdec1^-/-^* mice in Cage I compared to the *Adamdec1^-/-^* mice in Cage II (**Figure 5B**). The colon length did not differ significantly between the WT mice in Cage I and Cage II (**Figure 5B**). This mirrors the changes in weight in response to DSS observed across these four cohorts of mice.

### 3.5 Cage-dependent differences in colonic immune responses of *Adamdec1^−/−^* mice

**Figure 6:**
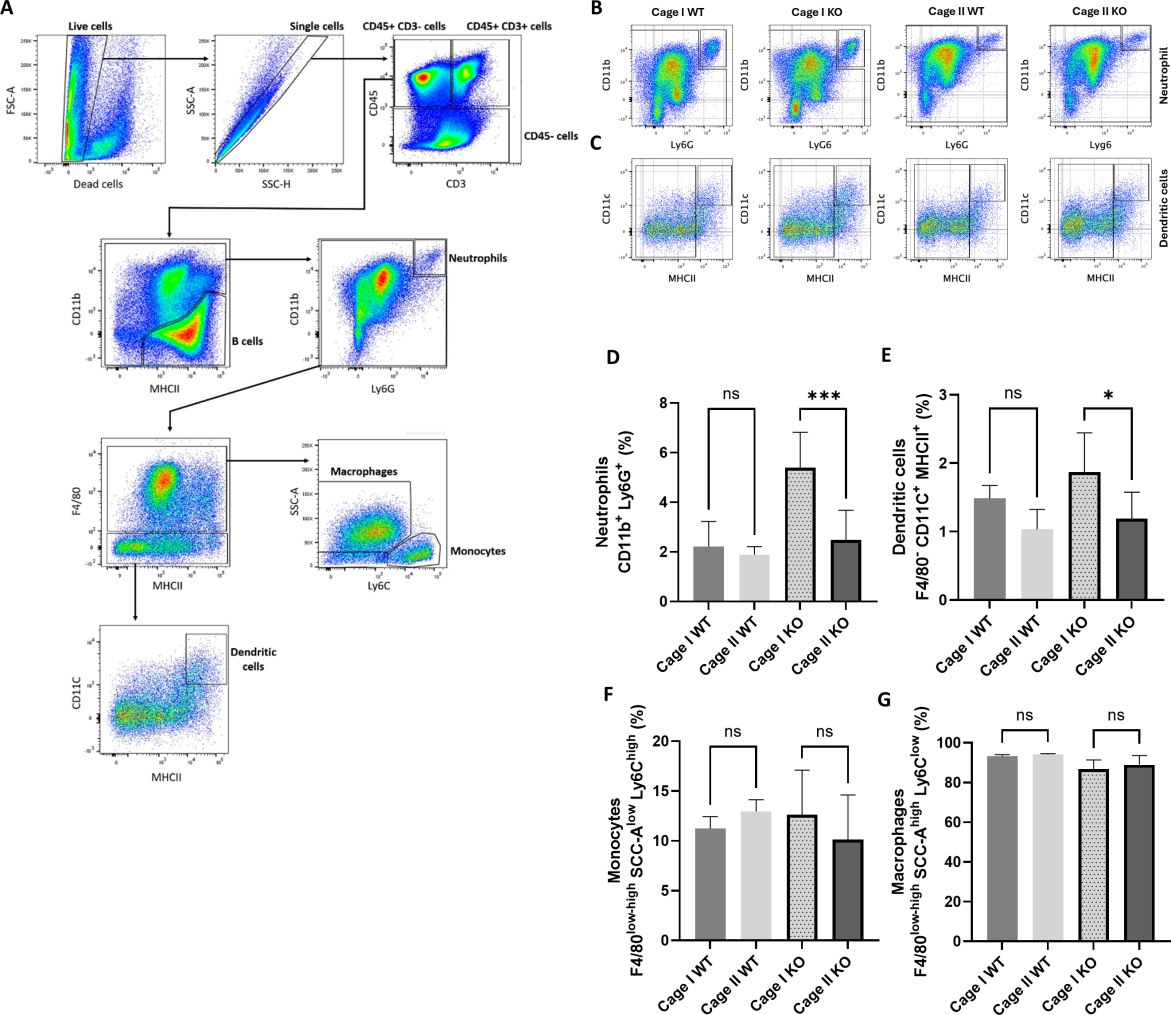
Cage-dependent alterations in colonic myeloid cell populations in DSS-treated *Adamdec1*^−/−^ and WT mice. Proportion of the myeloid lineage cells in the colonic tissues taken from *Adamdec1^-/-^* and WT mice in Cage I and Cage II on day 9 of 2% DSS challenge. **(A)** Gating strategy for myeloid cells in colons harvested from the mice, FACS plots of **(B)** neutrophil population (CD11b^+^ Ly6G^+^) and **(C)** dendritic cell (CD11c^+^ MHCII^+^) population in WT and *Adamdec1^-/-^* mice in Cage I and Cage II. Below, bar graphs show proportions of **(D)** Neutrophils, **(E)** Dendritic cells, **(F)** Monocytes, and **(G)** Macrophages. The proportions of neutrophils and dendritic cells were calculated from the CD45^+^ CD3− cell population after exclusion of B cells (CD45^+^ CD3− MHC^mid–high^). The proportions of monocytes and macrophages were calculated from the CD45^+^ CD3− F4/80^low–high^ cell population after exclusion of neutrophils (CD45^+^ CD3^-^ CD11b^+^ Ly6G^+^) and B cells. Data are shown as mean ± SD. Statistical comparisons were performed using one-way ANOVA. Cage I WT n = 3, Cage II WT n = 5, Cage I *Adamdec1^-/-^* n = 5, Cage II *Adamdec1^-/-^* n = 7. KO: *Adamdec1^-/-^*, *p < 0.05, ***p < 0.001, ns, Non-significant.

To investigate the differences in inflammatory responses between *Adamdec1^−/−^* mice in Cage I and Cage II, colons were harvested on day 9 of the DSS experiment, and colonic immune cell populations were analyzed by flow cytometry (**Figure 6A**).

The numbers of neutrophils and dendritic cells were significantly elevated in the colonic tissue of *Adamdec1^−/−^* mice from Cage I compared to their counterparts in Cage II (neutrophils *p* = 0.003, dendritic cells *p* = 0.033) (**Figures 6B–E**), consistent with the greater weight loss and shortened colon length observed in these animals (**Figures 5A, B**). In contrast, the numbers of monocytes and macrophages did not significantly differ between the knockout mice in Cage I and Cage II (**Figures 6F, G**). Notably, inflammatory cell populations did not differ significantly between WT mice from Cage I and Cage II following DSS treatment (**Figures 6B–G**).

**Figure 7:**
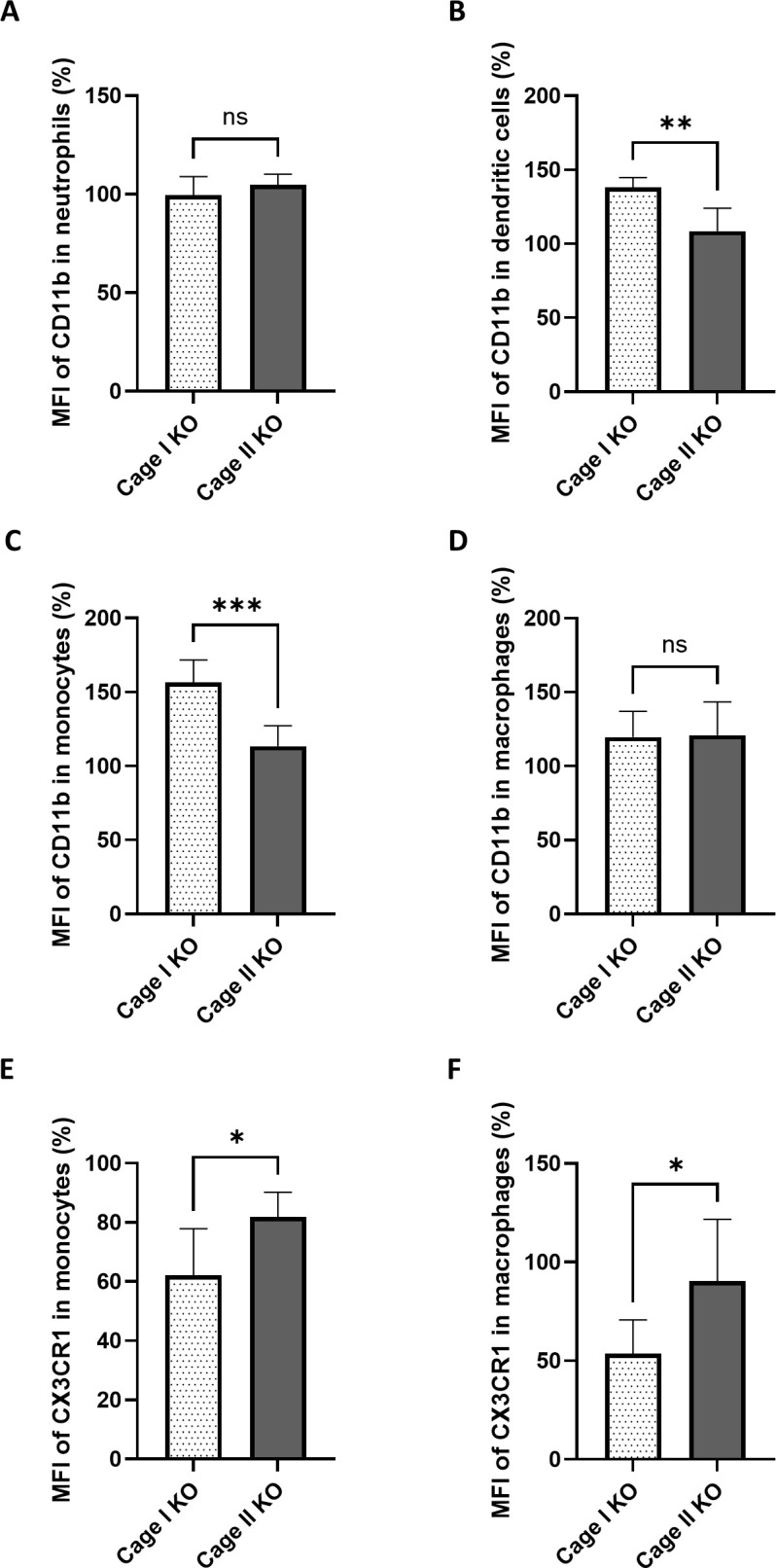
Cage-dependent differences in activation marker expression on colonic innate immune cells in DSS-treated *Adamdec1*^−/−^ mice. Activation markers on various innate inflammatory cells in the colonic tissues taken from *Adamdec1^-/-^* mice in Cage I and Cage II on day 9 of 2% DSS challenge. The mean fluorescence intensity (MFI) of each activation marker in each *Adamdec1^−/−^* sample was normalized to the mean MFI of WT samples for the corresponding activation marker within each cage. **(A)** CD11b on neutrophils (CD45^+^ CD3^-^ CD11b^+^ Ly6G^+^), **(B)** CD11b on dendritic cells (CD45^+^ CD3^-^ F4/80^-^ CD11c^+^ MHCII^+^), **(C)** CD11b on monocytes (CD45^+^ CD3^-^ F4/80^low-high^ SCC-A^low^ Ly6C^high^), **(D)** CD11b on macrophages (F4/80^low-high^ SCC-A^high^ Ly6C^low^), **(E)** CX3CR1 on monocytes, **(F)** CX3CR1 on macrophages. Data are shown as mean ± SD. Statistical comparisons were performed using two-tailed unpaired t-test. Cage I *Adamdec1^-/-^* n = 5, Cage II *Adamdec1^-/-^* n = 7. KO: *Adamdec1^-/-^*, *p < 0.05, **p < 0.01, ***p < 0.001, ns, Non-significant.

The activation states of myeloid cells of the *Adamdec1^-/-^* mice demonstrated an alteration based on cage and severity of the colitis (**Figure 7**). Specifically, *Adamdec1^−/−^* mice in Cage I exhibited higher surface expression of the activation marker CD11b on dendritic cells (*p* = 0.003) and monocytes (*p* = 0.0005) relative to *Adamdec1^−/−^* mice in Cage II (**Figures 7B, C**), indicative of heightened inflammatory activation. In contrast, expression of CX3CR1 (fractalkine receptor 1), a marker associated with anti-inflammatory signaling and maintenance of tissue homeostasis, was significantly increased on monocytes and macrophages from *Adamdec1^−/−^* mice in Cage II (*p* = 0.017 and *p* = 0.040, respectively) (**Figures 7E, F**). This expression pattern is consistent with the attenuated DSS-induced inflammatory response observed in these animals. For MFI-based analyses (**Figure 7**), WT animals were used as internal controls within each cage to account for batch effects. Accordingly, MFI values for*Adamdec1^-/-^* mice were normalized to their respective WT controls within the same cage. Thus, direct comparisons of WT MFI values between Cage I and Cage II are not presented.

### 3.6 Cage-dependent transcriptional responses to DSS in *Adamdec1^-/-^* mice

To further validate the differential response to DSS challenge observed between *Adamdec1^−/−^* mice in Cage I and Cage II, we assessed the expression of genes associated with inflammation and epithelial proliferation and repair.

**Figure 8:**
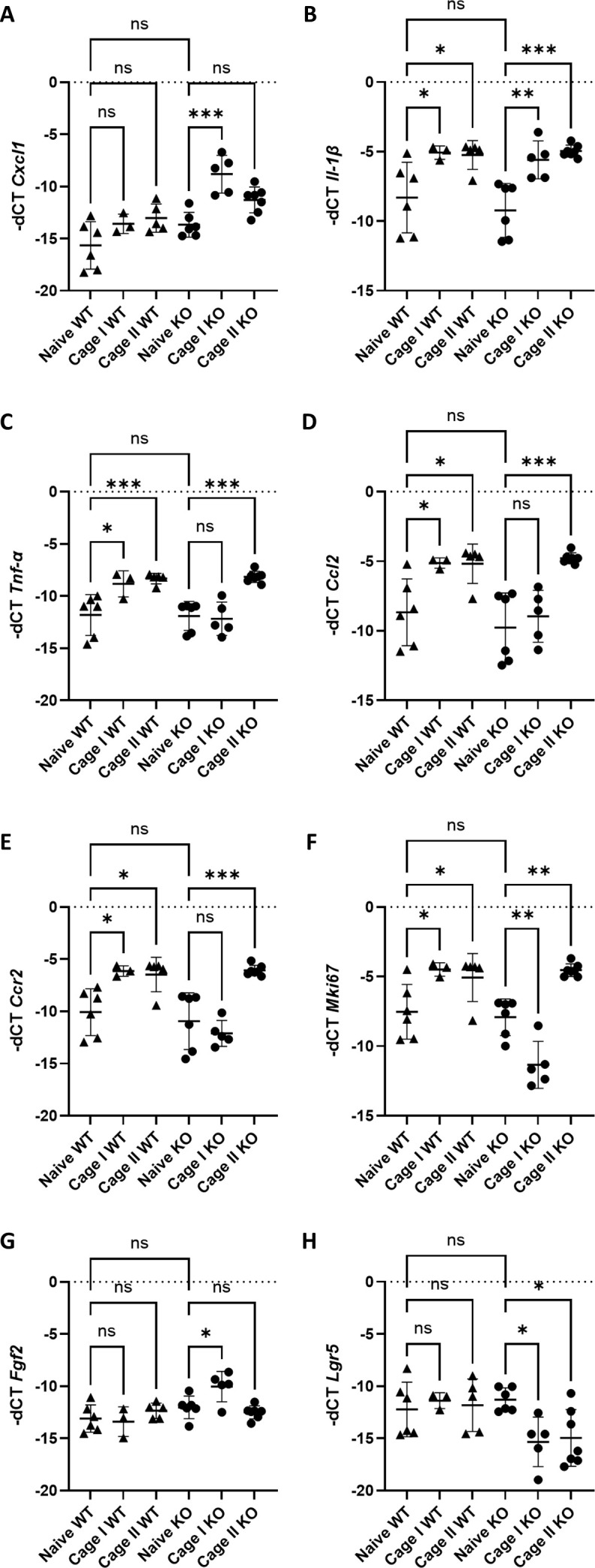
Differential expression of inflammatory and epithelial proliferation genes in DSS-treated and naïve mice. Change in expression of inflammatory and cell proliferation genes in colonic tissue from *Adamdec1^-/-^* and WT mice housed in Cage I or Cage II on day 9 of 2% DSS challenge, compared with naïve *Adamdec1^-/-^* and WT controls, quantified by qPCR. **(A)** *Cxcl1*, **(B)**, *Il-1β*, **(C)** *Tnf-α*, **(D)** *Ccl2*, **(E)** *Ccr2*, **(F)** *Mki67*, **(G)** *Fgf2*, and **(H)** *Lgr5* Data are shown as mean ± SD. Statistical comparisons were performed using one-way ANOVA. Naïve WT n = 6, naïve *Adamdec1^-/-^* n = 6. DSS Cage I WT n = 3, DSS Cage II WT n=5, DSS Cage I *Adamdec1-/-* n = 5, DSS Cage II *Adamdec1^-/-^* n = 7. KO: *Adamdec1^-/-^*, WT, Wild type, *p < 0.05, **p < 0.01, ***p < 0.001, ns, Non-significant.

At baseline, no significant differences in gene expression were observed between WT and *Adamdec1^−/−^* mice (**Figures 8A–H**). In WT mice, DSS challenge induced a robust and consistent inflammatory transcriptional response across cages, characterized by significant upregulation of *Il-1β* (*p* = 0.018), *Tnf-α* (*p* = 0.0002), *Ccl2* (*p* = 0.046), and *Ccr2* (*p* = 0.040), along with the elevation in the proliferation marker *Mki67* (*p* = 0.039) (**Figures 8B–F**). In contrast, expression of the neutrophil chemokine *Cxcl1*, epithelial repair–associated gene *Fgf2* and the intestinal stem cell marker *Lgr5* remained unchanged (**Figures 8A, G, H**). These DSS-induced transcriptional responses were consistent across cages in WT mice (**Figures 8A–H**).

In *Adamdec1^−/−^* mice, DSS-induced transcriptional responses were markedly cage dependent, with the exception of *Il1β*, and *Lgr5* (**Figures 8B, H**). The expression of *Il-1β* was significantly upregulated in *Adamdec1^−/−^* mice from both Cage I (*p* = 0.002) and Cage II (*p* = 0.0001) (**Figure 8B**).

In contrast, the expression of *Cxcl1* was significantly increased in *Adamdec1^−/−^* mice from Cage I (*p* = 0.0001) (**Figure 8A**), correlating with the increased weight loss, reduction in the colonic length (**Figures 5A, B**) and enhanced lamina propria neutrophil infiltration (**Figure 6A**) in these mice compared with *Adamdec1^−/−^* mice housed in Cage II. Furthermore, *Tnf-α* was significantly upregulated only in *Adamdec1^−/−^* mice from Cage II (*p* < 0.0001), mirroring the response observed in WT mice, but remained unchanged in *Adamdec1^−/−^* mice from Cage I despite their more severe inflammatory phenotype (**Figure 8C**). A similar pattern was observed for *Ccl2* and *Ccr2*, which were significantly induced in *Adamdec1^−/−^* mice within Cage II (*p* = 0.0002 and *p* = 0.0003, respectively), and in WT mice, but not in the knockout mice in Cage I (**Figures 8D, E**).

Markers of epithelial regeneration also displayed cage-specific regulation in the *Adamdec1^-/-^* mice. *Mki67* expression was significantly increased in *Adamdec1^−/−^* mice within Cage II following DSS treatment (*p* = 0.0008), consistent with initiation of epithelial repair and resembling the response observed in WT mice. In contrast, *Mki67* was significantly decreased in *Adamdec1^−/−^* mice within Cage I (*p* = 0.0016), indicative of impaired epithelial regeneration and greater mucosal damage (**Figure 8F**). *Fgf2* expression followed a similar pattern, being induced only in *Adamdec1^−/−^* mice housed in Cage I (*p* = 0.006) but remaining unchanged in *Adamdec1^−/−^* in Cage II and WT mice (**Figure 8G**).

Finally, the stem cell marker *Lgr5* was significantly downregulated following DSS challenge in *Adamdec1^−/−^* mice from both Cage I (*p* = 0.015) and Cage II (*p* = 0.027), while remaining unchanged in WT mice (**Figure 8H**). Notably, *Lgr5* was the only gene for which the DSS-induced response was consistent across *Adamdec1^−/−^* mice from the two separate cages, and this pattern diverged from that observed in WT mice.

Together, these findings support a role for ADAMDEC1 in regulating intestinal epithelial and inflammatory responses, and further suggest that environmental factors, likely mediated by the gut microbiome, contribute to differences in colitis severity in genetically susceptible animals.

### 3.7 Cage-dependent differences in gut microbiome composition of *Adamdec1^−/−^* mice

In contrast to WT mice, which exhibited a consistent and reproducible phenotypic response to DSS challenge, *Adamdec1^−/−^* mice displayed marked variability in disease severity that appeared to be influenced by environmental factors. To determine whether the gut microbiome contributed to this phenotypic variability, fecal samples collected following 8 weeks of cohousing in larger cages, prior to DSS administration, were analyzed for bacterial community composition.

**Figure 9:**
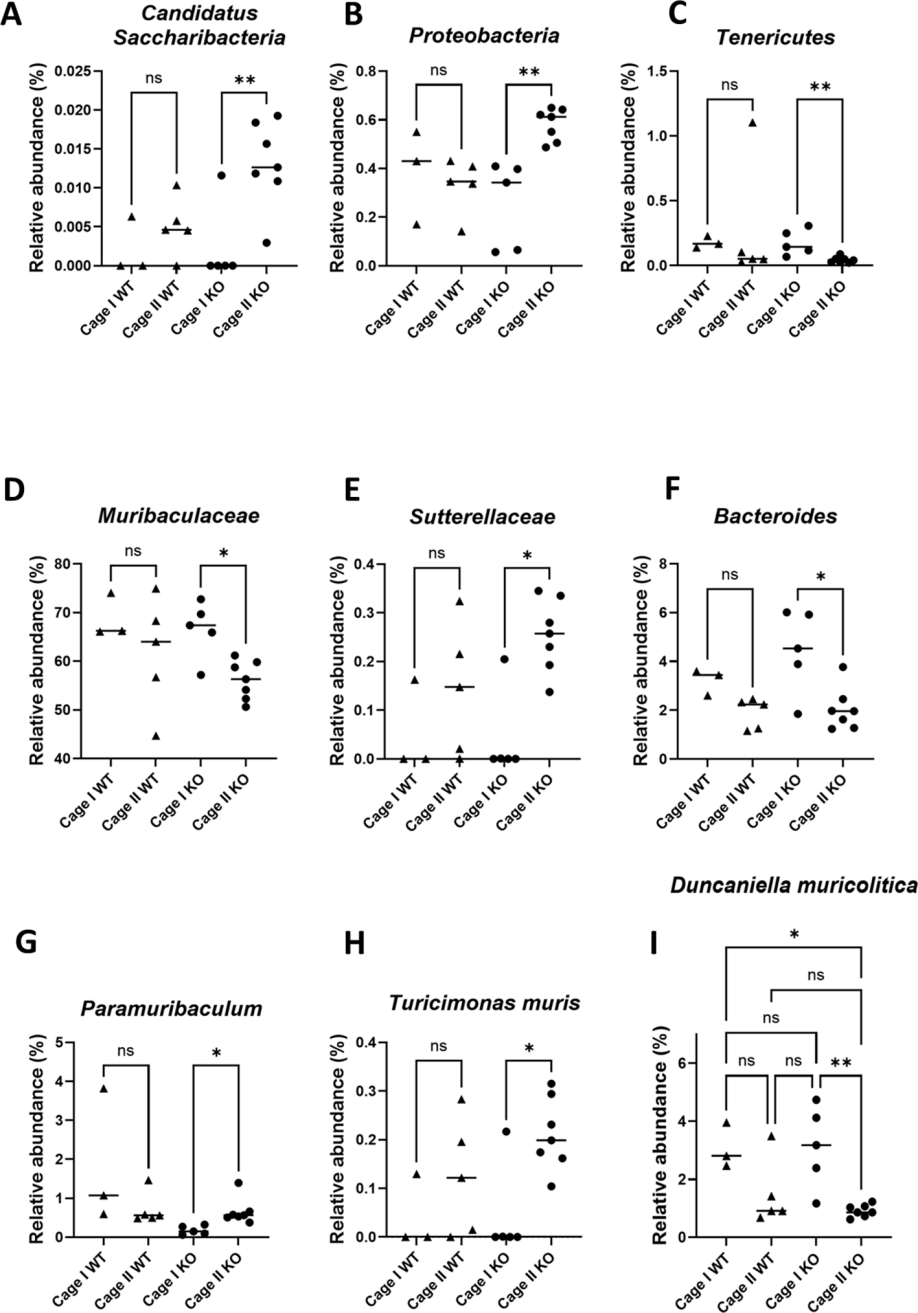
Cage-associated differences in fecal bacterial taxa following cohousing. Relative abundances of differentially abundant bacterial taxonomic groups in *Adamdec1^−/−^* and WT mice in Cage I and Cage II assessed at the phylum, family, genus and species level, using fecal samples collected after 8 weeks of cohousing prior to DSS challenge. **(A)** *Candidatus Saccharibacteria*, **(B)** *Proteobacteria*, **(C)** *Tenericutes*, **(D)** *Muribaculaceae*, **(E)** *Sutterellaceae*, **(F)** *Bacteroides*, **(G)** *Paramuribaculum* and **(H)** *Turicimonas muris*. Statistical comparisons were performed using Mann-Whitney test. **(I)** Relative abundance of *Duncaniella muricolitica* in *Adamdec1^-/-^* mice and WT mice in Cage I and Cage II collected after 8 weeks of cohousing prior to DSS challenge. Statistical comparisons were performed using Kruskal-Wallis test. Cage I WT n = 3, Cage II WT n = 5, Cage I *Adamdec1^-/-^* n = 5, Cage II *Adamdec1^-/-^* n = 7. KO: *Adamdec1^-/-^*, WT, Wild type; *p < 0.05, **p < 0.01; ns, Non-significant.

Significant differences in bacterial communities were observed between *Adamdec1^−/−^* mice from Cage I and Cage II. At the phylum level, *Candidatus Saccharibacteria* and *Proteobacteria* were significantly less abundant in Cage I compared with Cage II (0.002% vs. 0.01%, *p* = 0.007, and 0.25% vs. 0.58%, *p* = 0.001, respectively), whereas *Tenericutes* were significantly more abundant in cage I than in cage II (0.18% vs. 0.04%, *p* = 0.006) (**Figures 9A–C**).

At the family level, *Muribaculaceae* were significantly enriched in Cage I (64.90% vs. 56.86%, *p* = 0.017), while *Sutterellaceae* were significantly depleted (0.05% vs. 2.04%, *p* = 0.004) compared with Cage II (**Figures 9D, E**). At the genus level, *Bacteroides* was more abundant in Cage I (4.12% vs. 2.04%, *p* = 0.020), whereas *Paramuribaculum* was less abundant (0.15% vs. 0.47%, *p* = 0.018) compared with Cage II (**Figures 9F, G**).

Finally, at the species level, *Turicimonas muris* was significantly less abundant in Cage I than in Cage II (0.04% vs. 0.21%, *p* = 0.007) (**Figure 9H**).

We also examined the relative abundance of *Duncaniella muricolitica*, a bacterial species previously reported to exacerbate DSS-induced colitis (34). *D. muricolitica* was significantly enriched in *Adamdec1^−/−^* mice housed in Cage I compared with Cage II (3.12% vs. 0.91%, *p* = 0.002) (**Figure 9I**). Notably, the relative abundance of *D. muricolitica* was also significantly higher in the WT mice in Cage I than the knockout mice in Cage II (*p* = 0.023). This observation indicates that *D. muricolitica* alone does not drive severe DSS-induced colitis in WT mice but may exacerbate disease only in genetically susceptible hosts.

Collectively, these findings demonstrate that cage-dependent variation in gut microbiota composition precedes DSS challenge and correlates with divergent inflammatory outcomes in *Adamdec1^−/−^* mice, supporting a critical role for host–microbiome interactions in shaping disease severity.

## 4 Discussion

This study highlights the contributory role of environmental factors and gut microbial composition in modulating mucosal inflammation in genetically susceptible hosts. Our findings provide insight into how risk genotypes interact with the microbial communities to influence disease onset and severity in IBD and potentially help explain the marked clinical heterogeneity observed among affected individuals. Over the past few decades, considerable effort has been dedicated to identifying genetic risk factors for IBD, leading to the discovery of more than 300 disease-associated loci (35). However, these loci account for only a modest proportion of disease variance, which has largely been attributed to environmental influences that modulate genetic susceptibility. Evidence supporting this concept comes from studies involving monozygotic twins and migration cohorts, which consistently demonstrate the impact of non-genetic factors on disease expression (2–4, 6). While gene–environment interactions have been studied in cardiovascular, metabolic diseases and cancer, such investigations in IBD remain limited (36–38). Nonetheless, emerging evidence suggests that environmental exposures may directly influence IBD risk in a genotype-dependent manner (39, 40). Our findings extend this paradigm by demonstrating that loss of *Adamdec1* is associated with increased susceptibility to colitis in a manner that is influenced by environmental conditions. Specifically, we show that variation in colonic microbiome composition corresponds with divergent inflammatory outcomes in *Adamdec1-*deficient mice, despite genetic uniformity. These data identify ADAMDEC1 as a modifier of host–microbiome interactions and underscore the importance of environmental variability as a contributor to disease severity in genetically at-risk hosts.

We first demonstrated that the dominant factor shaping the murine gut microbiome was environment, consistently determined by cage, rather than genotype or sibling state, in accordance with previous studies (20, 33, 41). By co-housing genetically identical *Adamdec1^−/−^* and WT mice in two distinct environments, we found that environmental variation generated distinct microbial communities that in turn modulated inflammatory responses to DSS-induced colitis in *Adamdec1*-deficient mice. While under the conditions tested in this study WT mice exhibited consistent and reproducible responses across both cages, *Adamdec1^−/−^* mice displayed strikingly divergent outcomes, ranging from severe colitis in one environment to a mild, WT-like response in the other. These differences arose despite the use of highly controlled and standardised housing conditions, underscoring the significant influence of environmental factors on the penetrance and expression of a susceptible genotype.

The environment-associated divergence in colitis severity in *Adamdec1*^−/−^ mice was accompanied by marked differences in immune cell infiltration and activation. In mice from the severe-colitis environment, increased *Cxcl1* expression was observed compared with the mild-colitis environment, alongside elevated neutrophil infiltration and higher CD11b expression, consistent with enhanced CXCL1–CXCR2-driven recruitment and neutrophil-dominant inflammation (42, 43). Similarly, *Ccl2* and *Ccr2* expression were not elevated, and reduced *Cx3cr1* expression was observed in severe environment *Adamdec1^−/−^* mice, despite comparable monocyte and macrophage proportions across cages. *Cx3cr1* is expressed at high levels on patrolling monocytes and tissue-resident macrophages, where it promotes anti-inflammatory and homeostatic functions (44–46); its downregulation in Cage I *Adamdec1^−/−^* mice suggests a shift away from this regulatory phenotype and towards a more inflammatory myeloid cell state, potentially contributing to the amplified tissue damage observed in these animals. Notably, the absence of elevated *Tnf-α* and *Ccl2/Ccr2* in Cage I *Adamdec1^−/−^* mice, despite more severe disease, indicates a divergence from canonical monocyte-driven inflammatory signaling, and raises the possibility that the dominant inflammatory program in severe disease is neutrophil-led rather than monocyte-orchestrated. The shared *Il-1β* elevation across all groups and cages suggests this cytokine reflects a general response to DSS-induced mucosal injury rather than a driver of differential severity and is consistent with its established role as a broadly induced alarmin in intestinal inflammation. The precise role of IL-1β in colitis is still not fully elucidated and it has been shown that it can confer both damaging and protective effects (47–49). Together, these findings suggest that environment dependent differences in colitis severity are associated with distinct inflammatory programs, with a stronger contribution from neutrophil-associated chemokine signaling in severe disease. Further work is required to define the functional role and activation states of macrophages in this model, and in particular to determine whether the altered *Cx3cr1* expression reflects impaired monocyte-to-macrophage differentiation or a shift in macrophage polarization.

The gene expression data also reveal marked differences in epithelial integrity and regenerative capacity between *Adamdec1^−/−^* mice housed in the two environments. *Mki67*, a marker of cellular proliferation, was elevated in WT mice and Cage II knockouts, consistent with an active epithelial regenerative response to colitis induced injury. In contrast, Cage I knockout mice demonstrated a relative decrease in *Mki67* expression compared with naïve controls, suggesting a failure of the proliferative response and impaired crypt regeneration at the height of inflammation. This is consistent with the more severe macroscopic phenotype, greater weight loss and colon shortening, observed in this group, and may reflect epithelial exhaustion in the context of an exaggerated and poorly resolved inflammatory response. Reduced *Lgr5* expression in both knockout groups, irrespective of cage, points to a genotype-dependent loss of intestinal stem cell activity, suggesting that *Adamdec1* may play a role in maintaining the stem cell niche independently of the inflammatory environment. Previous studies have demonstrated a role for *Adamdec1* in colonic tissue remodeling and healing during colitis (30). ADAMDEC1 has also been shown to play an active role in stem cell self-renewal and maintenance in the context of glioblastoma (50). ADAMDEC1 was shown to function through the cleavage of FGF2 and the generation of a positive feedback loop which maintained stem cell self-renewal. Interestingly, within the context of colitis, *Fgf2* demonstrated a significant and selective elevation in Cage I knockout mice. FGF2, a pleiotropic growth factor induced under conditions of tissue stress and in addition to stem cell regulation, has also been reported in the context of mucosal wound healing (51–53). However, its elevation in the group with the most severe disease and impaired proliferation may reflect a compensatory but insufficient repair signal, or alternatively a role in the fibrotic and remodeling responses associated with severe colitis. Collectively, these data suggest that ADAMDEC1 deficiency predisposes to a failure of epithelial regeneration under inflammatory challenge, and that environmental factors further modulate this vulnerability, potentially through their effects on the intestinal microbiota and the inflammatory signals that shape the epithelial niche. Further work is required to define the functional and mechanistic role of ADAMDEC1 in the colonic inflammatory response during colitis.

Epithelial dysfunction further compounded disease severity in *Adamdec1^−/−^* mice. In the severe-colitis environment, *Mki67* expression was reduced following DSS challenge, indicating impaired epithelial proliferation and failure of mucosal repair. In addition, the expression of *Fgf2*, a key regulator of stem cell survival and epithelial homeostasis and repair, was increased in *Adamdec1^−/−^* mice from the severe environment, consistent with findings in IBD patient tissues and during DSS-induced colitis (51–53). By contrast, both WT mice and *Adamdec1*^−/−^ mice in the less severe environment exhibited increased *Mki67* expression, consistent with the initiation of epithelial regeneration. These findings suggest that either unchecked inflammation drives epithelial destruction or that *Adamdec1* deficiency intrinsically compromises epithelial repair in hostile environments. The consistent downregulation of the intestinal stem cell marker *Lgr5* across both environments in the absence of *Adamdec1* supports the latter possibility, pointing to a genotype-dependent vulnerability of the stem cell compartment to DSS-induced injury. In either case, the inability to restore the epithelial barrier likely perpetuates inflammation, creating a vicious cycle of tissue damage and immune activation. This interpretation aligns with previous findings in *Adamdec1^−/−^* mice exposed to DSS, which demonstrated increased susceptibility to epithelial injury, and identified *Adamdec1* expression in a subset of fibroblasts located at crypt edges, enriched for Wnt-regulating genes that are critical for stem cell proliferation, differentiation, and epithelial homeostasis (30). Loss of *Adamdec1* may, therefore, compromise Wnt-mediated support of the intestinal stem cell niche, weakening the regenerative capacity of crypts under inflammatory stress. Together, these findings highlight that *Adamdec1* deficiency, in combination with an adverse microbial environment, disrupts both epithelial barrier maintenance and stem cell–niche interactions, thereby amplifying disease severity.

The environment-dependent differences in colitis severity were strongly associated with microbiome composition in *Adamdec1^−/−^* mice. Cage-specific microbial communities differed substantially in *Adamdec1^−/−^* mice, with eight bacterial taxa showing significant differences in relative abundance between environments. The ameliorated degree of DSS-induced weight loss in the *Adamdec1^-/-^* mice housed in Cage II was consistent with previously reported homogenization of both genotype-specific microbiome and genotype-dependent difference in the severity of DSS-induced colitis by cohousing of WT and mutant mice (54–56). These findings suggest that environmental factors shaping microbial community structure may disproportionately affect genetically susceptible hosts compared with WT animals. However, the specific environmental drivers underlying the observed cage-dependent differences in microbiome composition remain unclear and warrant further investigation. Of particular interest, *D. muricolitica* - a species previously reported to exacerbate DSS colitis - was significantly enriched in both WT and *Adamdec1^-/-^* mice from the severe-colitis cage (34). This observation is important for two reasons. First, it reinforces that microbial composition was dictated primarily by environment rather than genotype. Second, it demonstrated that the pathogenic potential of *D. muricolitica* is genotype-dependent in which WT mice tolerated its presence without exacerbation of disease whereas *Adamdec1^−/−^* mice developed more severe colitis. These findings support the concept that certain microorganisms, such as *D. muricolitica*, act as conditional disease modifiers - benign in genetically resilient hosts but pathogenic in susceptible ones. This interpretation is further supported by our α-diversity findings, as co-housing reduced microbial diversity in Cage I but not Cage II. Notably, despite WT mice from Cage I exhibiting the greatest reduction in α-diversity and the lowest post-cohousing Chao1 index, which is typically associated with dysbiosis and increased inflammation (57, 58), these animals developed the mildest colitis.

This study has several limitations. Although ADAMDEC1 deficiency was confirmed by genotyping and loss of protein expression, whole-genome sequencing and formal off-target analyses were not performed. Therefore, the contribution of unidentified background genetic variants cannot be completely excluded. The experimental design did not include randomization at the level of individual animals during cage pooling, raising the possibility that pre-existing differences in the founding microbiota contributed to the observed outcomes. The overall sample size was modest and group sizes were unequal, which may limit statistical power, particularly for between-cage comparisons within genotype groups. Histological assessment of colonic tissue was not performed due to tissue constraints, as each colon was subdivided to enable downstream qPCR and flow cytometry analyses. As a result, direct evaluation of epithelial damage and inflammatory pathology at the tissue level was not possible. Initial DSS experiment included both male and female mice to assess potential sex-dependent effects, whereas the co-housing experiment was conducted in female-only cohorts due to animal welfare and housing constraints associated with group housing of adult males. Although no sex-dependent differences in DSS-induced colitis severity were observed in the initial experiment, a contribution of sex to microbiome composition or immune responses in the co-housing setting cannot be fully excluded. The present study used 16S rRNA gene sequencing for microbiota profiling, which has recognized constraints in taxonomic resolution at the species level and typically provides reliable classification only to the genus level. Consequently, species-level assignments reported here, should be interpreted with caution. Validation using species-specific qPCR or shotgun metagenomic sequencing, would be required to confirm these assignments. These considerations do not alter the broader conclusions drawn from genus- and family-level community analyses presented in this study, which are well within the resolving power of approaches based on 16S rRNA gene sequencing. Microbiome and inflammatory analyses are inherently correlative, and the absence of microbiota transfer or depletion experiments precludes definitive conclusions regarding causality. Finally, although co-housing provides a useful model to examine environmental influences, it does not fully disentangle the relative contributions of host genetics, microbial composition, and cage-specific factors. These limitations should be considered when interpreting the findings. Future studies incorporating dedicated tissue collection for histological assessment, inclusion of both sexes in co-housing designs, and mechanistic approaches to interrogate inflammatory pathways will be important to strengthen and extend these observations.

Despite these limitations, this study demonstrates that environmental and microbiome- associated factors are linked to differences in mucosal inflammation in genetically susceptible hosts, highlighting a gene–environment–microbiome axis that may provide a mechanistic basis for the clinical heterogeneity observed in patients with IBD. Further research into gene–environment interactions is needed not only to advance understanding of disease pathogenesis, but also to inform preventative, cost-effective, and personalized therapeutic strategies that reduce exposure to harmful environmental modifiers in genetically at-risk individuals.

## Data Availability

The datasets presented in this study can be found in online repositories. The accession number can be found below: https://www.ebi.ac.uk/ena, PRJEB109036.

## References

[1] Loddo I Romano C . Inflammatory bowel disease: genetics, epigenetics, and pathogenesis. Front Immunol. (2015) 6. doi: 10.3389/fimmu.2015.00551 26579126 PMC4629465

[2] Thompson NP Driscoll R Pounder RE Wakefield AJ . Genetics versus environment in inflammatory bowel disease: results of a British twin study. BMJ. (1996) 312:95–6. doi: 10.1136/bmj.312.7023.95 8555939 PMC2349773

[3] Halfvarson J Bodin L Tysk C Lindberg E Järnerot G . Inflammatory bowel disease in a Swedish twin cohort: a long-term follow-up of concordance and clinical characteristics. Gastroenterology. (2003) 124:1767–73. doi: 10.1016/S0016-5085(03)00385-8 12806610

[4] Ko Y Butcher R Leong R . Epidemiological studies of migration and environmental risk factors in the inflammatory bowel diseases. World J Gastroenterol. (2014) 20:1238. doi: 10.3748/wjg.v20.i5.1238 24574798 PMC3921506

[5] Yadav P Ellinghaus D Rémy G Freitag-Wolf S Cesaro A Degenhardt F . Genetic factors interact with tobacco smoke to modify risk for inflammatory bowel disease in humans and mice. Gastroenterology. (2017) 153:550–65. doi: 10.1053/j.gastro.2017.05.010 28506689 PMC5526723

[6] Orholm M Binder V Sørensen T Rasmussen L Kyvik K . Concordance of inflammatory bowel disease among Danish twins: results of a nationwide study. Scand J Gastroenterol. (2000) 35:1075–81. doi: 10.1080/003655200451207 11099061

[7] Durack J Lynch SV . The gut microbiome: relationships with disease and opportunities for therapy. J Exp Med. (2019) 216:20. doi: 10.1084/JEM.20180448 30322864 PMC6314516

[8] Gevers D Kugathasan S Denson LA Vázquez-Baeza Y Van Treuren W Ren B . The treatment-naive microbiome in new-onset Crohn’s disease. Cell Host Microbe. (2014) 15:382–92. doi: 10.1016/j.chom.2014.02.005 24629344 PMC4059512

[9] Joossens M Huys G Cnockaert M De Preter V Verbeke K Rutgeerts P . Dysbiosis of the faecal microbiota in patients with Crohn’s disease and their unaffected relatives. Gut. (2011) 60:631–7. doi: 10.1136/GUT.2010.223263 21209126

[10] Walker AW Sanderson JD Churcher C Parkes GC Hudspith BN Rayment N . High-throughput clone library analysis of the mucosa-associated microbiota reveals dysbiosis and differences between inflamed and non-inflamed regions of the intestine in inflammatory bowel disease. BMC Microbiol. (2011) 11:1–12. doi: 10.1186/1471-2180-11-7 21219646 PMC3032643

[11] Ding S Ma Y Liu G Yan W Jiang H Fang J . Lactobacillus brevis alleviates DSS-induced colitis by reprograming intestinal microbiota and influencing serum metabolome in murine model. Front Physiol. (2019) 10:1152. doi: 10.3389/fphys.2019.01152 31620010 PMC6759783

[12] Wang Y Xie Q Zhang Y Ma W Ning K Xiang JY . Combination of probiotics with different functions alleviate DSS-induced colitis by regulating intestinal microbiota, IL-10, and barrier function. Appl Microbiol Biotechnol. (2020) (1):335–49. doi: 10.1007/s00253-019-10259-6 31758237

[13] Zhu W Winter MG Byndloss MX Spiga L Duerkop BA Hughes ER . Precision editing of the gut microbiota ameliorates colitis. Nature. (2018) 553(7687):208–11. doi: 10.1038/nature25172 PMC5804340 29323293

[14] Perrone P D’Angelo S . Gut microbiota modulation through Mediterranean diet foods: implications for human health. Nutrients. (2025) 17:948. doi: 10.3390/nu17060948 40289944 PMC11944315

[15] Weersma RK Zhernakova A Fu J . Interaction between drugs and the gut microbiome. Gut. (2020) 69:1510–9. doi: 10.1136/gutjnl-2019-320204 32409589 PMC7398478

[16] Cázares-Olivera M Miroszewska D Hu L Kowalski J Jaakkola UM Salminen S . Animal unit hygienic conditions influence mouse intestinal microbiota and contribute to T-cell-mediated colitis. Exp Biol Med (Maywood). (2022) 247:1752–63. doi: 10.1177/15353702221113826 35946176 PMC9638955

[17] Galley JD Yu Z Kumar P Dowd SE Lyte M Bailey MT . The structures of the colonic mucosa-associated and luminal microbial communities are distinct and differentially affected by a prolonged murine stressor. Gut Microbes. (2014) 5:748–60. doi: 10.4161/19490976.2014.972241 25536463 PMC4615309

[18] Rothschild D Weissbrod O Barkan E Kurilshikov A Korem T Zeevi D . Environment dominates over host genetics in shaping human gut microbiota. Nature. (2018) 555:210–5. doi: 10.1038/nature25973 29489753

[19] Hildebrand F Nguyen TLA Brinkman B Yunta RG Cauwe B Vandenabeele P . Inflammation-associated enterotypes, host genotype, cage and inter-individual effects drive gut microbiota variation in common laboratory mice. Genome Biol. (2013) 14:1–15. doi: 10.1186/gb-2013-14-1-r4 23347395 PMC4053703

[20] McCafferty J Mühlbauer M Gharaibeh RZ Arthur JC Perez-Chanona E Sha W . Stochastic changes over time and not founder effects drive cage effects in microbial community assembly in a mouse model. ISME J. (2013) 7:2116–25. doi: 10.1038/ismej.2013.106 23823492 PMC3806260

[21] Ericsson AC Gagliardi J Bouhan D Spollen WG Givan SA Franklin CL . The influence of caging, bedding, and diet on the composition of the microbiota in different regions of the mouse gut. Sci Rep. (2018) 8:4065. doi: 10.1038/s41598-018-21986-7 29511208 PMC5840362

[22] Gregor A Fragner L Trajanoski S Li W Sun X Weckwerth W . Cage bedding modifies metabolic and gut microbiota profiles in mouse studies applying dietary restriction. Sci Rep. (2020) 10:20835. doi: 10.1038/s41598-020-77831-3 33257713 PMC7705694

[23] Zhang C Shi D Lai G Li K Zhang Y Li W . A transcriptome-wide association study integrating multi-omics bioinformatics and Mendelian randomization reveals the prognostic value of ADAMDEC1 in colon cancer. Arch Toxicol. (2025) 99:645–65. doi: 10.1007/s00204-024-03910-3 39680087

[24] Ku J Jeong E Gong JR Cho KH Sung CO Kim SH . Identification of a unique subpopulation of mucosal fibroblasts in colorectal cancer with tumor-restraining characteristics. Mol Cells. (2025) 48:100263. doi: 10.1016/j.mocell.2025.100263 40759242 PMC12419087

[25] O’Shea NR Chew TS Dunne J Marnane R Nedjat-Shokouhi B Smith PJ . Critical role of the disintegrin metalloprotease ADAM-like decysin-1 [ADAMDEC1] for intestinal immunity and inflammation. J Crohns Colitis. (2016) 10:1417–27. doi: 10.1093/ecco-jcc/jjw111 27226416 PMC5174729

[26] Mueller CG Rissoan MC Salinas B Ait-Yahia S Ravel O Bridon JM . Polymerase chain reaction selects a novel disintegrin proteinase from CD40-activated germinal center dendritic cells. J Exp Med. (1997) 186:655–63. doi: 10.1084/jem.186.5.655 9271581 PMC2199019

[27] Fritsche J Müller A Hausmann M Rogler G Andreesen R Kreutz M . Inverse regulation of the ADAM-family members, decysin and MADDAM/ADAM19 during monocyte differentiation. Immunology. (2003) 110:450–7. doi: 10.1111/j.1365-2567.2003.01754.x 14632642 PMC1783081

[28] De Schepper S Verheijden S Aguilera-Lizarraga J Viola MF Boesmans W Stakenborg N . Self-maintaining gut macrophages are essential for intestinal homeostasis. Cell. (2018) 175:400–415.e13. doi: 10.1016/j.cell.2018.07.048 30173915

[29] Kinchen J Chen HH Parikh K Antanaviciute A Jagielowicz M Fawkner-Corbett D . Structural remodeling of the human colonic mesenchyme in inflammatory bowel disease. Cell. (2018) 175:372–86. doi: 10.1016/j.cell.2018.08.067 30270042 PMC6176871

[30] Jasso GJ Jaiswal A Varma M Laszewski T Grauel A Omar A . Colon stroma mediates an inflammation-driven fibroblastic response controlling matrix remodeling and healing. PloS Biol. (2022) 20:e3001532. doi: 10.1371/journal.pbio.3001532 35085231 PMC8824371

[31] Li S Lu R Shu L Chen Y Zhao J Dai J . An integrated map of fibroblastic populations in human colon mucosa and cancer tissues. Commun Biol. (2022) 5:1326. doi: 10.1038/s42003-022-04298-5 36463319 PMC9719516

[32] Chew TS O’Shea NR Sewell GW Oehlers SH Mulvey CM Crosier PS . Optineurin deficiency contributes to impaired cytokine secretion and neutrophil recruitment in bacteria driven colitis. Dis Model Mech. (2015) 8(8):817–29. doi: 10.1242/dmm.020362 26044960 PMC4527293

[33] Lipinski JH Zhou X Gurczynski SJ Erb-Downward JR Dickson RP Huffnagle GB . Cage environment regulates gut microbiota independent of Toll-like receptors. Infect Immun. (2021) 89(9):e0018721. doi: 10.1128/IAI.00187-21 33941577 PMC8370678

[34] Forster SC Clare S Beresford-Jones BS Harcourt K Notley G Stares MD . Identification of gut microbial species linked with disease variability in a widely used mouse model of colitis. Nat Microbiol. (2022) 7:590–9. doi: 10.1038/s41564-022-01094-z 35365791 PMC8975739

[35] Liu Z Liu R Gao H Jung S Gao X Sun R . Genetic architecture of the inflammatory bowel diseases across East Asian and European ancestries. Nat Genet. (2023) 55:796–806. doi: 10.1038/s41588-023-01384-0 37156999 PMC10290755

[36] Dimou N Kim AE Flanagan O Murphy N Diez-Obrero V Shcherbina A . Probing the diabetes and colorectal cancer relationship using gene – environment interaction analyses. Br J Cancer. (2023) 129:511–20. doi: 10.1038/s41416-023-02312-z 37365285 PMC10403521

[37] Middha P Wang X Behrens S Bolla MK Wang Q Dennis J . A genome-wide gene-environment interaction study of breast cancer risk for women of European ancestry. Breast Cancer Res. (2023) 25:93. doi: 10.1186/s13058-023-01691-8 37559094 PMC10411002

[38] Sebastian SA Padda I Johal G . Cardiovascular-kidney-metabolic (CKM) syndrome: a state-of-the-art review. Curr Probl Cardiol. (2024) 49:102344. doi: 10.1016/j.cpcardiol.2023.102344 38103820

[39] Noble AJ Nowak JK Adams AT Uhlig HH Satsangi J . Defining interactions between the genome, epigenome, and the environment in inflammatory bowel disease: progress and prospects. Gastroenterology. (2023) 165:44–60.e2. doi: 10.1053/j.gastro.2023.03.238 37062395

[40] Bai J Bouwknegt DG Weersma RK Dijkstra G van der Sloot KWJ Festen EAM . Gene-environment interactions in inflammatory bowel disease: a systematic review of human epidemiologic studies. J Crohns Colitis. (2025) 19(6). doi: 10.1093/ecco-jcc/jjaf061 40464295 PMC12134891

[41] Singh G Brass A Cruickshank SM Knight CG . Cage and maternal effects on the bacterial communities of the murine gut. Sci Rep. (2021) 11:9841. doi: 10.1038/s41598-021-89185-5 33972615 PMC8110963

[42] Farooq SM Stillie R Svensson M Svanborg C Strieter RM Stadnyk AW . Therapeutic effect of blocking CXCR2 on neutrophil recruitment and dextran sodium sulfate-induced colitis. J Pharmacol Exp Ther. (2009) 329:123–9. doi: 10.1124/jpet.108.145862 19131582

[43] Pavlidis P Tsakmaki A Pantazi E Li K Cozzetto D Digby-Bell J . Interleukin-22 regulates neutrophil recruitment in ulcerative colitis and is associated with resistance to ustekinumab therapy. Nat Commun. (2022) 13:5820. doi: 10.1038/s41467-022-33331-8 36192482 PMC9530232

[44] Zigmond E Samia-Grinberg S Pasmanik-Chor M Brazowski E Shibolet O Halpern Z . Infiltrating monocyte-derived macrophages and resident Kupffer cells display different ontogeny and functions in acute liver injury. J Immunol. (2014) 193:344–53. doi: 10.4049/jimmunol.1400574 24890723

[45] Weber B Saurer L Schenk M Dickgreber N Mueller C . CX3CR1 defines functionally distinct intestinal mononuclear phagocyte subsets which maintain their respective functions during homeostatic and inflammatory conditions. Eur J Immunol. (2011) 41:773–9. doi: 10.1002/EJI.201040965 21341263

[46] Geissmann F Jung S Littman DR . Blood monocytes consist of two principal subsets with distinct migratory properties. Immunity. (2003) 19:71–82. doi: 10.1016/s1074-7613(03)00174-2 12871640

[47] Coccia M Harrison OJ Schiering C Asquith MJ Becher B Powrie F . IL-1β mediates chronic intestinal inflammation by promoting the accumulation of IL-17A secreting innate lymphoid cells and CD4(+) Th17 cells. J Exp Med. (2012) 209:1595–609. doi: 10.1084/jem.20111453 22891275 PMC3428945

[48] Liso M Verna G Cavalcanti E De Santis S Armentano R Tafaro A . Interleukin 1β blockade reduces intestinal inflammation in a murine model of tumor necrosis factor–independent ulcerative colitis. Cell Mol Gastroenterol Hepatol. (2022) 14:151–71. doi: 10.1016/j.jcmgh.2022.03.003 35314399 PMC9120241

[49] Aggeletopoulou I Kalafateli M Tsounis EP Triantos C . Exploring the role of IL-1β in inflammatory bowel disease pathogenesis. Front Med (Lausanne). (2024) 11:1307394. doi: 10.3389/fmed.2024.1307394 38323035 PMC10845338

[50] Jimenez-Pascual A Hale JS Kordowski A Pugh J Silver DJ Bayik D . ADAMDEC1 maintains a growth factor signaling loop in cancer stem cells. Cancer Discov. (2019) 9(11):1574–89. doi: 10.1158/2159-8290.CD-18-1308 31434712 PMC7400732

[51] Danopoulos S Schlieve CR Grikscheit TC Al Alam D . Fibroblast growth factors in the gastrointestinal tract: twists and turns. Dev Dyn. (2017) 246:344–52. doi: 10.1002/dvdy.24491 28198118

[52] Song X Dai D He X Zhu S Yao Y Gao H . Growth factor FGF2 cooperates with interleukin-17 to repair intestinal epithelial damage. Immunity. (2015) 43:488–501. doi: 10.1016/j.immuni.2015.06.024 26320657

[53] Houchen CW George RJ Sturmoski MA Cohn SM . FGF-2 enhances intestinal stem cell survival and its expression is induced after radiation injury. Am J Physiol. (1999) 276(1):G249–58. doi: 10.1152/ajpgi.1999.276.1.g249 9887002

[54] Nunberg M Werbner N Neuman H Bersudsky M Braiman A Ben-Shoshan M . Interleukin 1α-deficient mice have an altered gut microbiota leading to protection from dextran sodium sulfate-induced colitis. mSystems. (2018) 3(3):e00213-17. doi: 10.1128/mSystems.00213-17 29766049 PMC5940968

[55] Elinav E Strowig T Kau AL Henao-Mejia J Thaiss CA Booth CJ . NLRP6 inflammasome regulates colonic microbial ecology and risk for colitis. Cell. (2011) 145:745–57. doi: 10.1016/j.cell.2011.04.022 21565393 PMC3140910

[56] Brinkman BM Becker A Ayiseh RB Hildebrand F Raes J Huys G . Gut microbiota affects sensitivity to acute DSS-induced colitis independently of host genotype. Inflammation Bowel Dis. (2013) 19:2560–7. doi: 10.1097/MIB.0b013e3182a8759a 24105395

[57] Gong D Gong X Wang L Yu X Dong Q . Involvement of reduced microbial diversity in inflammatory bowel disease. Gastroenterol Res Pract. (2016) 2016:6951091. doi: 10.1155/2016/6951091 28074093 PMC5198157

[58] Abdel-Rahman LIH Morgan XC . Searching for a consensus among inflammatory bowel disease studies: a systematic meta-analysis. Inflammation Bowel Dis. (2023) 29:125–39. doi: 10.1093/ibd/izac194 36112501 PMC9825291

